# Anti-erbB2 treatment induces cardiotoxicity by interfering with cell survival pathways

**DOI:** 10.1186/bcr1523

**Published:** 2006-07-13

**Authors:** Thea Pugatsch, Suzan Abedat, Chaim Lotan, Ronen Beeri

**Affiliations:** 1 The Cardiovascular Research Center, Heart Institute, Hadassah-Hebrew University Medical Center, Jerusalem, Israel

## Abstract

**Introduction:**

Cardiac dysfunction is among the serious side effects of therapy with recombinant humanized anti-erbB2 monoclonal antibody. The antibody blocks ErbB-2, a receptor tyrosine kinase and co-receptor for other members of the ErbB and epidermal growth factor families, which is over-expressed on the surface of many malignant cells. ErbB-2 and its ligands neuregulin and ErbB-3/ErbB-4 are involved in survival and growth of cardiomyocytes in both postnatal and adult hearts, and therefore the drug may interrupt the correct functioning of the ErbB-2 pathway.

**Methods:**

The effect of the rat-anti-erbB2 monoclonal antibody B-10 was studied in spontaneously beating primary myocyte cultures from rat neonatal hearts. Gene expression was determined by RT-PCR (reverse transcription polymerase chain reaction) and by rat stress-specific microarray analysis, protein levels by Western blot, cell contractility by video motion analysis, calcium transients by the FURA fluorescent method, and apoptosis using the TUNEL (terminal uridine nick-end labelling) assay.

**Results:**

B-10 treatment induces significant changes in expression of 24 out of 207 stress genes analyzed using the microarray technique. Protein levels of ErbB-2, ErbB-3, ErbB-4 and neuregulin decreased after 1 day. However, both transcription and protein levels of ErbB-4 and gp130 increased several fold. Calreticulin and calsequestrin were overexpressed after three days, inducing a decrease in calcium transients, thereby influencing cell contractility. Apoptosis was induced in 20% cells after 24 hours.

**Conclusion:**

Blocking ErbB-2 in cultured rat cardiomyocytes leads to changes that may influence the cell cycle and affects genes involved in heart functions. B-10 inhibits pro-survival pathways and reduces cellular contractility. Thus, it is conceivable that this process may impair the stress response of the heart.

## Introduction

Human epidermal growth factor receptor (Her)-2 (ErbB-2), a 185 kDa transmembrane glycoprotein receptor with intrinsic tyrosine kinase activity, is thought to be one of the important mediators of cell growth [1-5]. It is overexpressed in 25–30% of human breast cancers, plays a role in its pathogenesis and is a predictive marker for poor prognosis in patients with metastatic disease [6-10].

Over recent years new therapies were developed to target tumour cells with Her-2 overexpression by blocking Her-2 on the cell surface of the tumour cells, thereby inhibiting their growth. The most widely used drug based on this principle is Trastuzumab (Herceptin^®^, Genentech Inc., San Francisco, CA, USA), a high-affinity humanized anti-Her-2 antibody that was shown to benefit patients with Her-2-overexpressing breast cancer, either as a single agent or in combination with chemotherapy [6,11,12]. In women who had received at least one prior chemotherapeutic regimen for metastatic disease, the response rate was found to be 15% and in previously untreated patients 23%.

One of the major side effects noted was cardiotoxicity [6]. Congestive heart failure linked to Herceptin therapy may be severe and has been associated with disabling symptoms. Of patients receiving Herceptin together with paclitaxel, 12% developed cardiac dysfunction, as compared with 1% receiving paclitaxel alone; the number increased further when Herceptin was administered in combination with anthracyclines (27%) compared with anthracyclines alone (7%) [6,13]. No data are yet available on identification of patients who are at risk for developing cardiac dysfunction when they receive anti-erbB2 therapy, although both prior cardiotoxic therapy (e.g. radiation to the breast area) and advanced age may play a role.

ErbB-2 forms heterodimers with other ErbB receptors (ErbB-3/ErbB-4) that can bind neuregulins, polypeptide growth factors that are known to promote survival and growth of cardiac myocytes [14,15]. Thus, blocking ErbB-2 with a monoclonal antibody may inhibit the myocardial adaptation to physiological stress and injury, such as that resulting from chemoradiotherapy, ultimately leading to cardiac failure in susceptible patients.

In order to elucidate the molecular mechanisms that lead to cardiotoxocity, we studied the effect of the rat-anti-erbB2 monoclonal antibody B-10 on spontaneously beating primary cultures derived from rat neonatal hearts. B-10 has previously been shown to have activity biologically similar to that of the humanized antibody that is used in the clinical setting [16].

## Materials and methods

### Tissue culture

Neonatal rat ventricular muscle cells were prepared and plated as described by Landau and coworkers [17]. Briefly, ventricles from one-day-old rats were minced and treated with trypsin, and the combined fractions were resuspended in growth medium (Ham's F10 containing 10% foetal calf serum, 10% horse serum, 200 U/ml penicillin, 200 μg/ml streptomycin, 135 mg/ml CaCl_2 _and 1 mmol/l glutamine; all from Biological Industries, Kibbutz Bet Haemek, Israel) in a sterile flask (Nunc; Nuclon Delta Herlev, Denmark) through a sterile mesh to exclude explants. The pooled cells were suspended in growth medium to a density of 9 × 10^5 ^to 1 × 10^6 ^cells/ml and seeded into 30 mm diameter, six-well dishes (Falcon Labware, Oxnard, CA, USA). After 24–36 hours, this concentration yielded a near confluent layer of beating heart cells at a final density of about 2 × 10^6 ^cells/ml. Cultures were kept at 37°C in an atmosphere of 5% carbon dioxide and 95% air. Experiments were performed at day 6, when more than 80% of the culture consisted of myocardial cells. Cultures were serum starved for 24-hours before addition of the monoclonal antibody. (The investigation conforms to the *Guide for the Care and Use of Laboratory Animals*, published by the US National Institutes of Health [18]).

### B-10

Rat c-erbB-2/HER-2/neu Ab-9 (Neomarkers, Fremont, CA, USA) was added at different concentrations (1.25 μg/ml to 7.5 μg/ml), to determine the optimal conditions by which the cells will retain their structure and continue beating. Viability was determined by Trypan blue exclusion analysis. The concentration of 5 μg/ml B-10 was chosen for all experiments; treated cells were incubated at 37°C in an atmosphere of 5% carbon dioxide and 95% air for 24 hours. Cells were harvested with trypsin/EDTA and RNA was prepared using a commercially available kit (Boehringer Mannheim-Roche, Basel, Switzerland).

### Reverse transcription polymerase chain reaction

The primers used in the study are summarized in Table 1.

### Microarrays

To analyze gene expression, we used the Atlas™ Nylon cDNA Rat stress Expression array (Clontech Pharmingen, Palo Alto, CA, USA), containing 207 genes, in accordance with the manufacturer's instructions. Analysis of the results was carried out using software provided by Clontech.

### Western blot analysis

Polyclonal antibodies to ErbB-2, ErbB-3, ErbB-4, neuregulin, calreticulin, calsequestrin, gp130, akt, p-akt, β-actin and the secondary antibodies were purchased from Santa Cruz Biotechnology Inc. (Santa Cruz, CA, USA) and Jackson Laboratories (Bar Harbor, Maine, USA) SDS-PAGE, transfer and analysis were carried out in accordance with standard procedures [19]. Briefly, proteins were separated on 5–15% SDS-polyacrylamide gradient gels, and transferred to nitrocellulose membranes using a Hoeffer transfer chamber. The membranes were blocked with either 0.5% donkey serum or 2% skim milk, depending on the antibody. Bands were detected after incubation with Western Blotting Luminol reagent (Santa Cruz Biotechnology Inc.).

### Cell contractility

Cultured cardiomyocytes were placed on the temperature-controlled stage of an inverted microscope coupled to a video-motion analyzer; cells were paced at a constant rate to control for independent effects of changes in the beating rate. Analysis was performed after marking manually the poles of one cell pacing at 60/min; contractility is expressed as percentage shortening.

### Calcium transients

Calcium transients were determined by a modification to the FURA fluorescence method [20].

### TUNEL assay

TUNEL (terminal uridine nick-end labelling) assay was carried out using the Promega DeadEnd™ Fluorometric TUNEL system, in accordance with the manufacturer's instructions, based on the method described by Ben-Sasson and coworkers [21]. Slides were analyzed with a confocal laser scanning microscope (Zeiss 410, Carl Zeiss GmbH, Goettingen Germany). More than 1500 nuclei were counted per field; the experiment was repeated three times.

## Results

### Tissue culture

The effect of B-10 on the morphology of neonatal rat ventricular heart muscle cells *in vitro *was determined by adding the antibody at concentrations ranging between 1.25 μg/ml and 7.5 μg/ml. After 24 hours cells were analyzed visually. Between concentrations of 1.25 μg/ml and 5 μg/ml, no difference in morphology and spontaneous beating could be observed, whereas at concentrations higher than 5 μg/ml cells appeared round and increased in size, no sharp intact cell membrane could be detected and most cells had died. At 5 μg/ml B-10 cells retained their size and shape, but they seemed to beat more slowly compared with untreated control cells. Cell viability, as determined by Trypan blue exclusion analysis, was 89%, which is comparable to that of untreated control cells; therefore, the concentration of 5 μg/ml was chosen for all subsequent experiments.

### Gene expression

Of the 207 genes tested, the expression of 24 was modulated by the antibody (Table 2). Most prominently affected were proteins involved in the cell cycle, a number of the heat shock proteins and proteins involved in calcium exchange.

**Figure 1:**
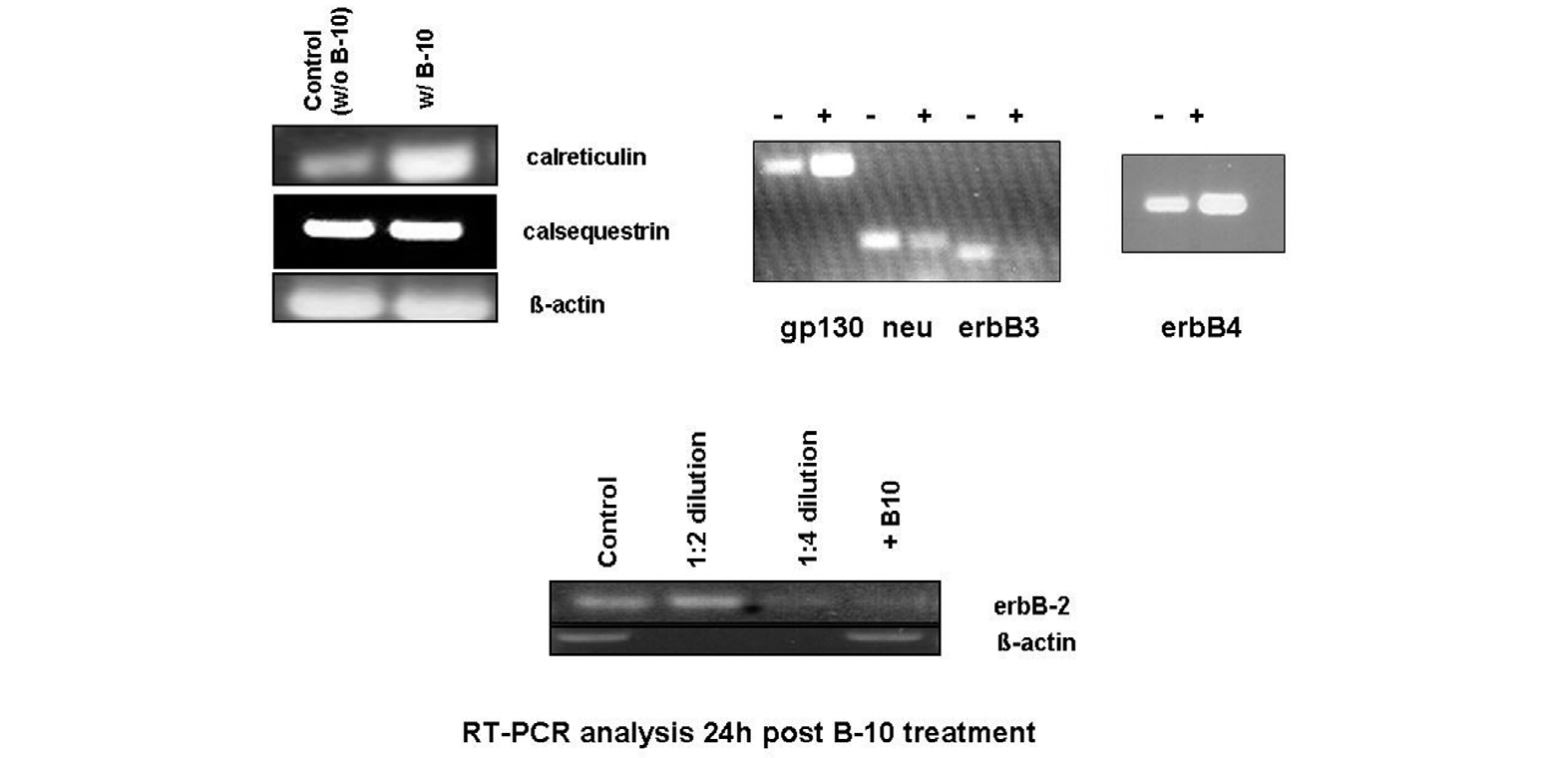
RT-PCR analysis of control and treated (B-10) cells. Primers used are described in Table 1. RT-PCR, reverse transcription polymerase chain reaction.

**Figure 2:**
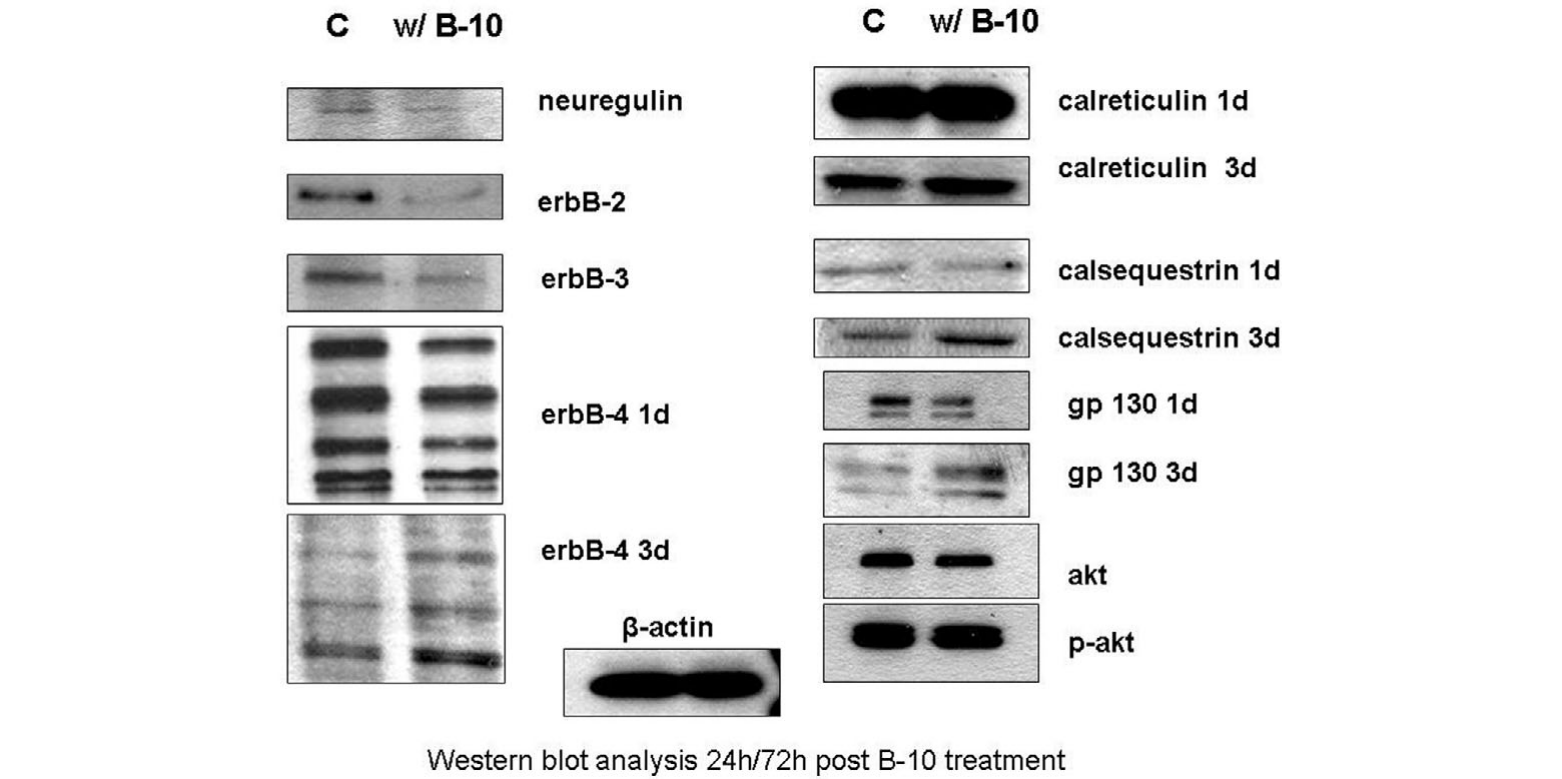
Western blot analysis of control and treated (B-10) cells after 24/72 hours.

We further analyzed different members of the ErbB family, two known ligands for ErbB-2 (neuregulin and gp130), and calreticulin and calsequestrin using both RT-PCR (Figure 1) and Western blot analyses (Figure 2).

Twenty-four hours after incubation, B-10 substantially downregulated the transcription of ErbB-2 (fourfold) as well as that of ErbB-3, whereas transcription of ErbB-4 was increased (Figure 2); ErbB-2 itself, as expected, as well as both ErbB-3 and ErbB-4 were downregulated at the translational levels (Table 3).

In the microarray analysis, we found that calreticulin, a calcium storage protein in the sarcoplasmatic reticulum, was upregulated. Although this protein is necessary for heart development in the embryo, it is usually not expressed in the adult cell [22]. As shown in Figure 1, upon administration of B-10 calreticulin transcription increased threefold. No change could be detected at the protein level after one day; however, after three days its level increased compared with that in control cells (Figure 2). Whereas no effect on both calsequestrin transcription and translation was detected after one day, protein levels were higher after three days (Figures 1 and 2, Table 3).

We then analyzed two additional ligands of ErbB-2, namely neuregulin and gp130. Both transcription and translation of neuregulin were downregulated nearly twofold, whereas gp130 mRNA levels increased at least threefold. The protein levels of neuregulin are consistent with the RT-PCR finding. Surprisingly, hardly any difference could be detected in the protein levels of gp130; in fact, we noticed a slight decrease (Figures 1 and 2, Table 3).

As representatives of the cell survival pathway, we chose to determine the levels of akt-1 and p-akt-1 in B-10 treated cardiomyocytes. As shown in Figure 2, no effect was seen on p-akt-1, whereas akt-1 expression was slightly but not significantly lower than that of control cells.

### Cell contractility and calcium transients

**Figure 3:**
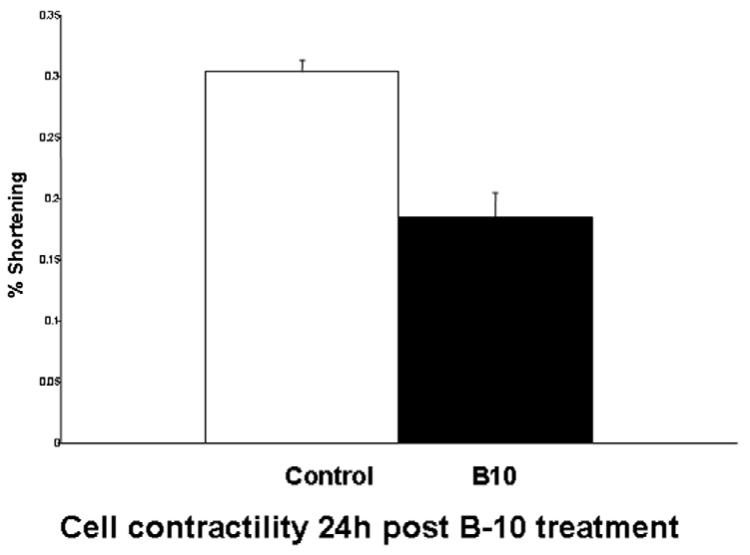
Contraction, as assessed by percentage shortening. The values (mean ± standard deviation) are as follows: control 0.3037 ± 0.0096 and B-10 treatment 0.1842 ± 0.0205. *P *< 0.001.

**Figure 4:**
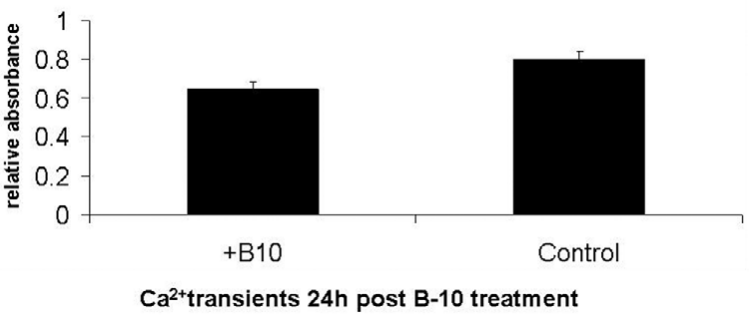
Calcium transient analysis. The values (relative absorbance; mean ± standard deviation) are as follows: control 0.803 ± 0.04 and B-10 0.644 ± 0.04. *P *< 0.001.

The antibody severely interferes with cell contractility, which was reduced by 40% compared with that in control cells (Figure 3). The difference in calcium transients between treated and control cells reached 20% (Figure 4).

### Apoptosis

**Figure 5:**
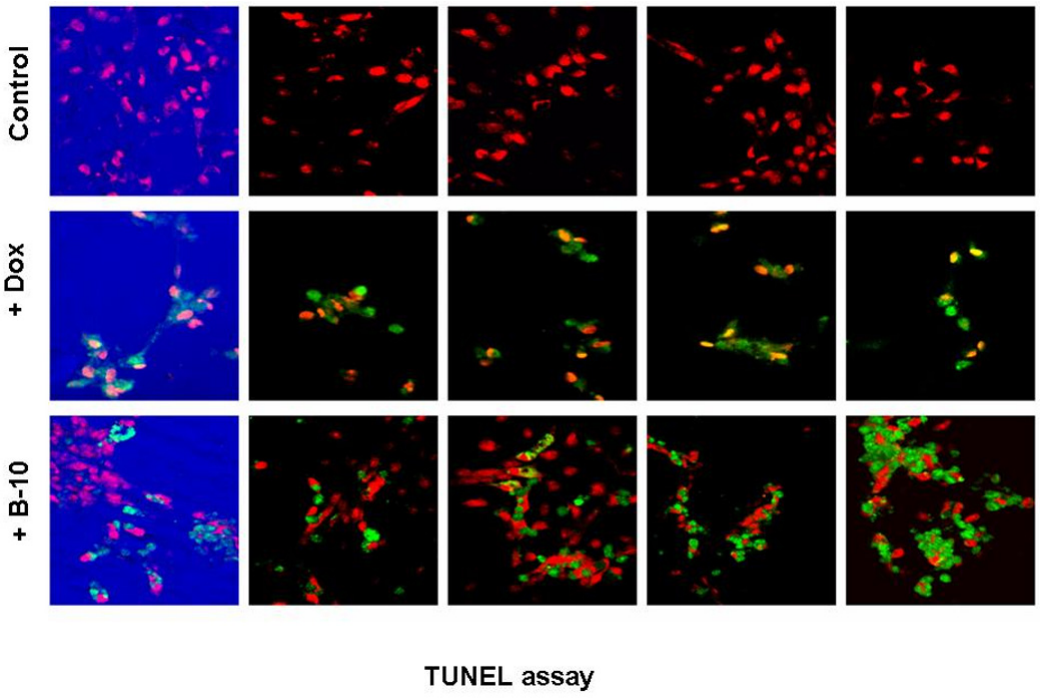
TUNEL assay, 24 hours after addition of B-10 or doxorubicin. The first row shows untreated control cells. The second row shows doxorubicin (10 μmol/l) treated positive control cells. The third row shows B-10 treated cells. TUNEL, terminal uridine nick-end labeling.

**Figure 6:**
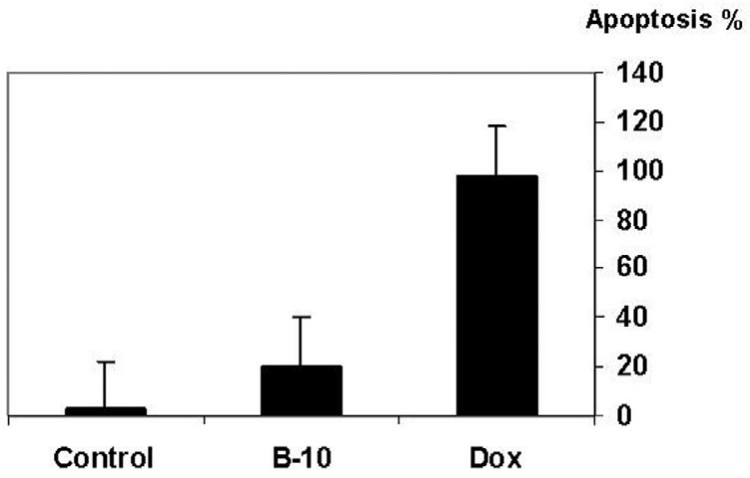
Percentage of apoptotic cells 24 hours after addition of B-10 or doxorubicin. The TUNEL method was used to determine the percentage of apoptotic cells. The values (mean ± standard deviation) are as follows: control cells 2.35 ± 0.02%; doxorubicin treated cells 98 ± 0.09%; and B-10 treated cells 20.4 ± 0.09%.

Both treated cells and control cultures were analyzed for induction of apoptosis 24 hours after adding B-10 or doxorubicin (10 μmol/l). B-10 treated cells underwent rapid apoptosis (20 ± 0.09%) compared with doxorubicin-treated positive control cells (98 ± 0.09%), and very little apoptosis in untreated control cells (2.35 ± 0.02%; Figures 5 and 6).

## Discussion

Targeted monoclonal antibody therapy as a biological treatment for different cancers appears to have many advantages over conventional chemo/radiotherapy. In the case of ErbB-2 overexpressing breast cancer cells, it was postulated that antibody therapy would specifically eliminate only tumour cells without affecting any other ErbB-2 expressing cells [23-25]. Although erbB-2 is expressed ubiquitously, it was thought that those levels are sufficiently low not to be affected by anti-erbB-2 antibodies. However, ErbB-2 plays a vital role both in developing and in adult murine cardiomyocytes [14,26-28]. ErbB-2 null mutant mice exhibit an absence of trabecules in the heart ventricle and die at midgestation [14]. Adult mice carrying a conditional ErbB-2 mutation develop severe dilated cardiomyopathy, usually in the second month of life [28]. As shown by Özcelik and coworkers [29], loss of ErbB-2 in cardiomyocytes leads to physiological stress on the heart, which over time induces cardiac decompensation and dilated cardiomyopathy.

The effect of trastuzumab on cells on the molecular level has repeatedly been analyzed on human breast cancer cell lines [30,31]. However, to our knowledge very few molecular studies on the effect of the antibody on cardiomyocytes have been reported. *In vitro *cultures of primary human cardiomyocytes are not readily attainable for both ethical and technical reasons. Therefore, in our study we opted to use the generally accepted *in vitro *model of neonatal rat ventricular heart muscle cells. Trastuzumab, being a humanized monoclonal antibody, does not bind to rat cells, consequently we used B-10 instead, which was shown previously to have biologic activity similar to that of trastuzumab [16,32]. Our results reveal a substantial decrease in the level of all ErbB family members analyzed after treatment with B-10. ErbB-2 by itself has no tyrosine kinase activity and needs to form heterodimers with one of its ligands to be functional [33]. All the ligands tested in our system were downregulated, thereby affecting the central role played by ErbB-2 in the heart. Moreover, gp130, which is thought to bind to ErbB-2 after biomechanical stress (hypoxia, haemodynamic overload and myocardial injury, among other insults) and activates a survival pathway [34], is strongly affected by B-10. Our results imply that the turnover of gp130 is augmented, as reflected in the several fold increase in transcription; nevertheless, the decrease in gp130 cannot be completely compensated.

It was previously demonstrated that overexpression of calreticulin modulates protein kinase B/Akt signalling, promoting apoptosis during cardiac differentiation of cardiomyoblasts [35]. In our system B-10 induced an increase in both calreticulin and calsequestrin levels three days after antibody treatment. The high levels of both of these calcium storage proteins in the sarcoplasmic reticulum may modify calcium release and subsequently lead to reduced cell contractility, decreasing the percentage shortening by 40%. This finding corroborates the studies described in ErbB-2 mutant mice and calsequestrin overexpressing mice [36,37]. Sawyer and coworkers [38], when comparing anthracycline- and ErbB2-induced myofibrillar disarray, reported no effect of B-10 on either akt or p-akt in cultures of adult rat ventricular myocytes, which is consistent with our data.

Finally, apoptosis may be a factor in the loss of ventricular function that is part of the cardiotoxic side effect profile of anti-erbB2 monoclonal antibody therapy. In our system B-10-induced apoptosis was three times greater than that recently described by Grazette and coworkers [39]. However, those investigators used a different antibody (clone 9G6), which may account for the difference in intensity.

## Conclusion

In this molecular analysis of the effects of B-10 on neonatal cardiomyocytes, we demonstrated that the rat-anti-erbB2 monoclonal antibody B-10 severely affects ErbB-2 and its major ligands ErbB-3/4, neuregulin and gp130. In addition, the drug promotes increased expression of calreticulin and calsequestrin, which are involved in the sarcoplasmatic reticulum calcium pump and decrease cell contractility, which *in vivo *may lead to dilated cardiomyopathy in mice and humans as described previously [22,28,34,40]. Despite being present at a low level, in cardiomyocytes ErbB-2 plays a central role in the correct functioning of the cell. Blocking ErbB-2 by a monoclonal antibody disrupts the cell survival pathways and the ErbB-signalling network that depends on the relative stoichiometry between all of the players involved. We therefore speculate that this process may impair the stress response of the heart.

## Abbreviations

RT-PCR, reverse transcription polymerase chain reaction.

## Competing interests

The authors declare that they have no competing interests.

## Authors' contributions

TP conceived the study, participated in its design and coordination, and wrote the manuscript. SA conducted all of the laboratory experiments. CL participated in discussions. RB participated in the design study. All authors read and approved the final manuscript.

## Figures and Tables

**Table 1:** Primers used in the study

Gene	Primers
ErbB-2	5'-TGGTGGTCGTTGGAATCCTAATC-3' 5'-CCAGCCATCACATACGCTTCATC-3'

ErbB-3	5'-GGTGCCAAAGGTCCAATCTA C-3' 5'-ACCGTCACCGCTATTACCAG-3'

ErbB-4	5'-CACAACCAGCACCATACCAG-3' 5'-TTCATCCAGTTCTGCTCGTG-3'

Neuregulin	5'-GAAGGCGCAAACACTTCTTC-3' 5'-TTGGCAACGATCACCAGTAA-3'

gp130	5'-TGAAGACAGACCATCCAAAGCG-3' 5'-CCATTCTACCCAGAGCAGGTTATC-3'

Calreticulin	5'-TCCCTGACCCTGATGCTAAGAAG-3'
	
	5'-ATTGTGCCAGACTTGACCTGCC-3'

Calsequestrin	5'-CCAGAGCATCTGTGGACTCA-3' 5'-AGCCAGCAGACCTGAACAGT-3'

β-actin	5'-TTGTAACCAACTGGGACGATATGG-3' 5'-GATCTTGATCTTCATGGTGCTAGG-3'

**Table 2:** Genes that are either upregulated or downregulated by B-10

Gene	Up/down^a^
Mitogen-activated protein kinase 9 (*MAPK9*; *PRKM9*); c-jun N-terminal kinase 2 (*JNK2*); stress-activated protein kinase alpha (*SAPK*-α)	**↑**
Heat shock 27-kDa protein (*HSP27*)	**↑**
Heat shock 90-kDa protein beta (*HSP90*-β); *HSP84*; *HSPCB*	**↑**
Heat shock 60-kDa protein (*HSP60*); 60-kDa chaperonin (*CPN60*); GroEL homolog; mitochondrial matrix protein P1; p60 lymphocyte protein	**↑**
Heat shock 47-kDa protein (*HSP47*); collagen-binding protein 1 (*CBP1*)	**↓**
HSP70/HSP90-organizing protein (*HOP*); p60 protein	**↓**
Heat shock 10-kDa protein (*HSP10*); chaperonin 10 (*CPN10*)	**↓**
p23; 23-kDa progesterone receptor-associated protein (rat homologue of human)	**↓**
Vimentin (*VIM*)	**↑**
T-complex protein 1 alpha subunit (*TCP1-α*); CCT-alpha (*CCTA*; *CCT1*)	**↓**
T-complex protein 1 eta subunit (*TCP1-eta*); CCT-eta (*CCTH*; *CCT7*); HIV-1 NEF interacting protein (rat homologue of human)	**↑**
Calreticulin (*CALR*); calregulin; calcium-binding protein 3 (*CABP3*); *HACBP*; *ERP60*	**↑**
Endoplasmic reticulum stress protein 72 (*ERP72*); calcium-binding protein 2 (*CABP2*)	**↓**
M-phase inducer phosphatase 2 (*MPI2*); cell division control protein 25 B (*CDC25B*)	**↓**
G_1_/S-specific cyclin D_2 _(*CCND2*); vin-1 proto-oncogene	**↑**
G_1_/S-specific cyclin D_3 _(*CCND3*)	**↓**
G_2_/M-specific cyclin G (*CCNG*)	**↑**
Inhibitor of DNA binding 1 (*ID1*)	**↑**
Inhibitor of DNA binding 2 (*ID2*)	**↓**
Prothymosin-alpha (*PTMA*)	**↑**
Soluble superoxide dismutase 1 (*SOD1*)	**↓**
DNA topoisomerase I (*TOP1*; rat homologue of human)	**↑**
Nucleophosmin (*NPM*); nucleolar phosphoprotein B23; numatrin; nucleolar protein NO38	**↓**
Ribosomal protein S19 (*RPS19*)	**↓**

^a^Upregulated (↑) or downregulated (↓).

**Table 3:** Summary of RT-PCR and Western blot analysis

	RT-PCR	Western blot	
		
		1 day	3 days
ErbB-2	↓	↓	ND
ErbB-3	↓	↓	ND
ErbB-4	↑	↓	ND
Calreticulin	↑	=	↑
Calsequestrin	=	=	↑
Gp130	↑	↓	ND
Neu	↓	↓	ND
Akt	ND	=	ND
p-Akt	ND	=	ND

↑, upregulated; ↓, downregulated; =, no change; ND, not determined; RT-PCR, reverse transcription polymerase chain reaction.
